# The Early Safety Signal of Sacituzumab Govitecan-Related Toxicity and the UGT1A1*28 Genotype in Metastatic Breast Cancer: A Real-World Preliminary Report

**DOI:** 10.3390/jcm15051715

**Published:** 2026-02-24

**Authors:** María Martínez-Pérez, María Teresa Nieto-Sánchez, Xando Díaz-Villamarín, Alicia Torres-García, Emilio Fernández-Varón, Alvaro Prados-Carmona, Marta Legerén, José Cabeza-Barrera, Isabel Blancas, Rocío Morón

**Affiliations:** 1 Hospital Pharmacy, Hospital Universitario San Cecilio, 18016 Granada, Spain; mariamape30@gmail.com (M.M.-P.); maite.nieto.mn@gmail.com (M.T.N.-S.); jose.cabeza.sspa@juntadeandalucia.es (J.C.-B.); 2 Instituto de Investigación Biosanitaria de Granada (Ibs.Granada), 18012 Granada, Spain; alicia.torres@genyo.es (A.T.-G.); emiliofv@ugr.es (E.F.-V.); apradoscar@gmail.com (A.P.-C.); marta.legeren@gmail.com (M.L.); iblancas@ugr.es (I.B.); 3 Department of Pharmacology and Therapeutics, Faculty of Medicine, Autonomous University of Madrid, 28029 Madrid, Spain; 4 Department of Pharmacology, Center for Biomedical Research (CIBM), University of Granada, 18016 Granada, Spain; 5 Department of Dermatology, Hospital Universitario San Cecilio, 18016 Granada, Spain; 6 Medical Oncology, Hospital Universitario San Cecilio, 18016 Granada, Spain

**Keywords:** sacituzumab-govitecan, UGT1A1*28, pharmacogenetics, antibody–drug conjugates, SN-38

## Abstract

**Background/Objectives:** Sacituzumab govitecan (SG) releases SN-38, the same active metabolite as irinotecan, thereby sharing key metabolic pathways and toxicity mechanisms. The clearance of SN-38 is strongly influenced by *UGT1A1* polymorphisms, particularly the UGT1A1*28 allele. While UGT1A1*28 genotyping routinely guides irinotecan dosing, no such recommendations exist for SG. This study describes the relationship between UGT1A1*28 and severe SG-related toxicity in real-world practice, identifying early safety signals and exploring the clinical and economic impact. **Methods:** This retrospective observational study (2021–2025) included patients with metastatic breast cancer treated with SG and patients with advanced gastrointestinal malignancies treated with irinotecan at a tertiary hospital. In the SG cohort, genotyping followed grade ≥3 toxicity; in the irinotecan cohort, it was performed prospectively. Toxicity (Common Terminology Criteria for Adverse Events version 5.0) and healthcare costs related to hospitalizations were estimated using official institutional tariffs. **Results:** All nine SG patients with severe toxicity (100%) carried the UGT1A1*28 allele. In the irinotecan cohort (n = 74), which was managed with genotype-guided dosing, severe toxicity and hospitalization were less frequent. SG was associated with higher mean costs per treated patient (€2817.01 vs. €1233.63), driven by toxicity-related admissions (33.3% vs. 10.8%). Genotyping costs (€10.51) were negligible compared to daily hospitalization expenses (up to €1984.90). **Conclusions:** Severe SG-related toxicity reveals a consistent UGT1A1*28-associated vulnerability. Given the drug’s recent approval in Spain, these data represent an urgent real-world safety signal. The marked disparity between low genotyping costs and high hospitalization expenses supports implementing preventive *UGT1A1* testing to optimize the safety and sustainability of sacituzumab govitecan therapy.

## 1. Introduction

Triple-negative metastatic breast cancer (mTNBC) is one of the most aggressive and lethal subtypes of breast cancer, characterised by marked biological heterogeneity, a lack of hormone receptor and human epidermal growth factor receptor 2 (HER2) expression, rapid disease progression, and limited availability of effective therapeutic options. Unlike other breast cancer subtypes, patients with mTNBC cannot benefit from endocrine- or anti-HER2-targeted therapies, making cytotoxic chemotherapy and, more recently, antibody–drug conjugates (ADCs) the mainstay of treatment. In recent years, ADCs have emerged as one of the most innovative therapeutic strategies in oncology, demonstrating promising efficacy across multiple solid tumours, including breast cancer [1,2,3,4].

**Figure 1:**
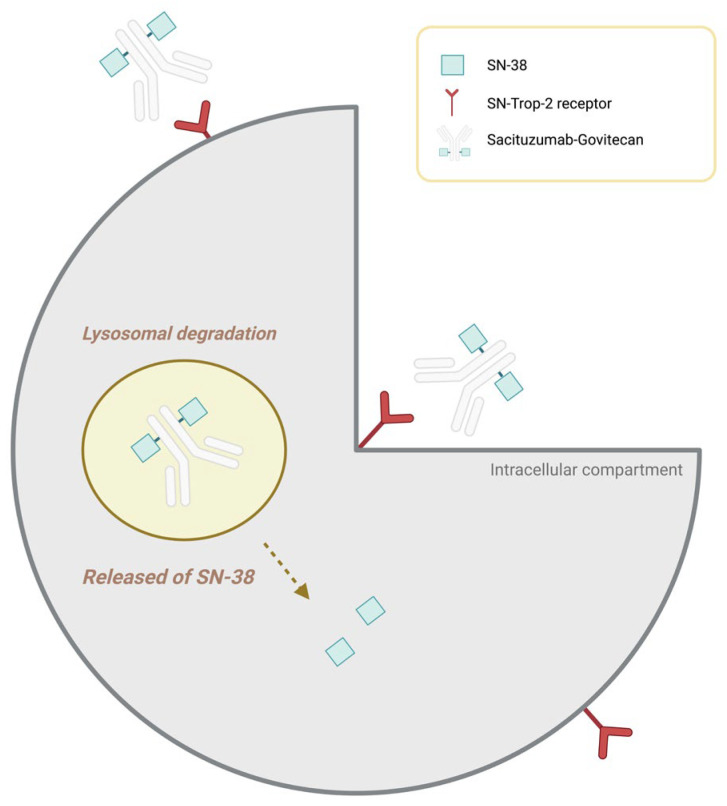
Mechanism of SN-38 release from sacituzumab govitecan (SG). SG is an antibody–drug conjugate directed against the Trop-2 receptor, which is overexpressed in various epithelial tumours. Following binding to Trop-2 on the tumour cell surface, the SG–Trop-2 complex is internalised and undergoes lysosomal degradation, resulting in intracellular release of the active metabolite SN-38, which mediates the cytotoxic effect. “Created in BioRender.com”.

In this context, sacituzumab govitecan (SG) has represented a significant advance in the treatment of metastatic breast cancer, both in the mTNBC subtype and in hormone receptor–positive, HER2-negative (HR+/HER2−) disease. SG is an antibody–drug conjugate targeting trophoblast cell-surface antigen 2 (Trop-2), a transmembrane glycoprotein widely expressed in epithelial tumours, including both subtypes of advanced breast cancer. Through a hydrolysable linker, the antibody is conjugated to SN-38, the active metabolite of irinotecan and a potent topoisomerase I inhibitor, enabling selective intracellular release of the cytotoxic payload following internalisation and lysosomal degradation of the antibody–receptor complex (Figure 1) [5].

SG has been shown to significantly improve progression-free survival and overall survival in patients with mTNBC, as demonstrated in the ASCENT trial [6], and in pretreated HR+/HER2− breast cancer, as shown in the TROPiCS-02 trial [7], leading to its regulatory approval in both indications. According to its summary of product characteristics, SG is indicated for the treatment of patients with unresectable or metastatic disease who have received multiple prior lines of systemic therapy, including endocrine therapy in the HR+/HER2− subtype [8].

**Figure 2:**
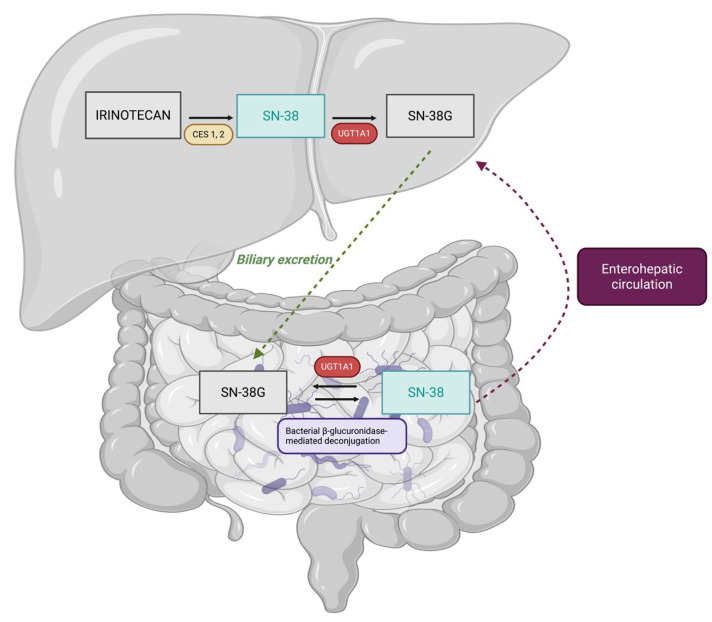
Metabolism of irinotecan. Irinotecan is administered as a prodrug and is converted to SN-38, the active metabolite responsible for both antitumour efficacy and toxicity, by hepatic carboxylesterases (CES1 and CES2). SN-38 is subsequently inactivated through glucuronidation mediated by uridine diphosphate glucuronosyltransferase 1A1 (UGT1A1), forming SN-38 glucuronide (SN-38G), an inactive metabolite that is predominantly excreted via the bile. “Created in BioRender.com”.

Given that the cytotoxic payload released by SG is SN-38, the same active metabolite of irinotecan, its pharmacokinetic behaviour and toxicity profile are closely linked to the well-characterised metabolic pathways of irinotecan. Therefore, a review of irinotecan metabolism is essential to contextualise the potential role of the UGT1A1 gene in SG-associated toxicity. The pharmacology of irinotecan and its relationship with SN-38 have been extensively studied over the past few decades. Following administration, irinotecan acts as a prodrug that requires metabolic activation by hepatic carboxylesterases (CES1 and CES2) to generate SN-38, the metabolite responsible for both antitumour efficacy and dose-limiting haematological and gastrointestinal toxicities. SN-38 is subsequently detoxified almost exclusively through glucuronidation mediated by uridine diphosphate glucuronosyltransferase 1A1 (UGT1A1), producing the inactive metabolite SN-38 glucuronide (SN-38G), which is eliminated primarily via the biliary route and, to a lesser extent, through renal excretion (Figure 2) [9].

It has been robustly demonstrated that UGT1A1 activity exhibits substantial genetically determined interindividual variability. The UGT1A1*28 polymorphism, caused by the presence of an additional TA repeat in the promoter region of the gene (from TA6 to TA7), is associated with a marked reduction in gene expression and enzymatic activity [10,11]. Consequently, the capacity to glucuronidate SN-38 is diminished, leading to increased systemic exposure to this active metabolite and a higher risk of severe toxicity, particularly grade 3–4 neutropenia and severe diarrhea [12,13,14]. In light of this robust body of evidence, both the U.S. Food and Drug Administration [15] and the European Medicines Agency [16] recommend dose reductions in irinotecan in patients who are homozygous for UGT1A1*28, especially when high-dose regimens are used. Likewise, several pharmacogenetic guidelines, including those from the Clinical Pharmacogenetics Implementation Consortium (CPIC) [17], the Spanish Society of Pharmacogenetics and Pharmacogenomics (SEFF) [18], and the Dutch Pharmacogenetics Working Group (DPWG) [19], support the use of *UGT1A1* genotyping prior to the initiation of irinotecan treatment in order to anticipate toxicity and guide dose adjustment. Clinical studies have demonstrated that this pharmacogenetic-guided strategy significantly reduces the incidence of severe irinotecan-associated toxicity [20].

Although SG is an antibody–drug conjugate, several pharmacokinetic studies have demonstrated that its administration is associated with clinically relevant systemic exposure to the active metabolite SN-38. Following SG infusion, measurable plasma concentrations of free SN-38 are detected, with area under the concentration–time curve (AUC) values and maximum plasma concentrations (C_max) that may be comparable to, or even exceed, those observed after conventional irinotecan administration. This systemic exposure results not only from intracellular release following internalisation of the antibody–receptor complex but also from extracellular release of SN-38 mediated by the hydrolysable linker, contributing to the so-called bystander effect. Consequently, a clinically relevant fraction of SN-38 reaches the systemic circulation and must be eliminated through the same metabolic pathways as irinotecan-derived SN-38, primarily via *UGT1A1*-dependent hepatic glucuronidation. These findings challenge the assumption that antibody–drug conjugates inherently limit systemic exposure to their cytotoxic payload and suggest that genetic determinants of SN-38 clearance may also play a clinically relevant role in patients treated with SG [21].

Although SG shares the same active metabolite as irinotecan, SN-38, and its administration is associated with clinically relevant systemic exposure to this metabolite, there are currently no formalised therapeutic recommendations based on *UGT1A1* genotype for this drug. The available evidence regarding the influence of *UGT1A1* genotype on SG-associated toxicity remains limited and is derived mainly from exploratory analyses and observational studies with small sample sizes. Although the official product information for Trodelvy acknowledges an increased risk of neutropenia and other adverse reactions in UGT1A1*28 carriers, current clinical guidelines do not recommend routine genotyping or genotype-guided dose adjustments for patients treated with SG [22]. However, accumulating experience from real-world clinical practice and data from observational studies suggest that toxicity associated with SG may be influenced by *UGT1A1* genotype, particularly in carriers of the *28 allele. Early reports, including post hoc analyses of pivotal clinical trials, indicated higher rates of severe neutropenia among patients homozygous for UGT1A1*28, although statistical significance was not consistently achieved, likely due to limited sample sizes [5,23]. More recent real-world studies have demonstrated a consistent trend toward a higher incidence of grade ≥3 toxicity in UGT1A1*28 carriers, including heterozygous individuals [23]. Despite this growing body of evidence, these findings have not translated into regulatory updates, and SG continues to be administered without dose adjustment or specific pharmacogenetic-based recommendations [24].

The discrepancy between the robust pharmacogenetic evidence supporting irinotecan and the absence of comparable recommendations for SG is particularly striking, given that both drugs rely on the same active metabolite, share identical elimination pathways, and display overlapping toxicity profiles [25]. However, the strength of evidence underpinning these regulatory approaches differs substantially. For irinotecan, decades of research have firmly established the *UGT1A1* genotype as a clinically relevant predictor of toxicity [19]. In contrast, for SG, the evidence remains emergent and limited, with methodological constraints related to small sample sizes, retrospective study designs, and heterogeneity in genetic characterisation across studies [22,26].

Adding to this uncertainty is the fact that SG is used in a particularly vulnerable population, such as heavily pretreated patients with mTNBC, who typically have a narrow therapeutic margin and a high risk of grade ≥3 toxicity [8]. In this context, the identification of reliable predictors of toxicity is essential to optimise clinical management and to prevent early treatment discontinuation, dose reductions, or toxicity-related hospitalisations, all of which may adversely affect patients’ quality of life and clinical outcomes.

From an economic perspective, severe SN-38 related toxicity represents a substantial healthcare burden, largely driven by the management of febrile neutropenia, toxicity-related hospital admissions, the use of broad-spectrum antibiotics and colony-stimulating factors, as well as treatment delays or interruptions, all of which are associated with significant increases in healthcare costs [27]. Although the cost of *UGT1A1* genotyping is considerably lower than that of a single hospital admission, its implementation in the context of SG has not been widespread due to the absence of robust evidence supporting pharmacogenetic-guided dose adjustments [28]. In this setting, evaluating the role of genotype as a predictor of toxicity and estimating the economic impact of incorporating pharmacogenetics into SG prescribing represent key steps toward advancing personalised medicine in the field of antibody–drug conjugates.

Analysing SN-38-associated toxicity across two drugs that share the same active metabolite, one with established pharmacogenetic-guided dose adjustment strategies (irinotecan) and another without specific therapeutic recommendations (SG), allows the clinical expression of toxicity to be contextualised under different pharmacogenetic management scenarios. This comparative approach facilitates the exploration of a potential pharmacogenetic signal for SG in real-world clinical practice and, although it does not establish causal relationships, may contribute to hypothesis generation and inform the design of prospective studies aimed at optimising treatment safety.

The aim of this study was to describe the relationship between the UGT1A1*28 genotype and the occurrence of severe SG-associated toxicity in real-world clinical practice to contextualise these findings using a cohort of patients treated with irinotecan in whom pharmacogenetic-guided dose adjustment strategies are applied and to explore the clinical and economic impact of such toxicity.

## 2. Materials and Methods

### 2.1. Study Design

A retrospective observational study was conducted at a tertiary care hospital and included patients treated with SG or irinotecan between October 2021 and July 2025. All participants underwent *UGT1A1* genotyping to determine *28 allele status, although the timing and clinical indication for testing differed between cohorts. In the irinotecan group, genotyping was performed prospectively prior to treatment initiation in accordance with standard clinical practice. In the SG cohort, genotyping was performed retrospectively and exclusively in patients who developed grade ≥3 toxicity, introducing a selection bias toward poorer tolerance that should be taken into account when interpreting toxicity outcomes. This approach reflects real-world clinical practice, as no genotype-guided dosing recommendations are currently available for SG and treatment is initiated at a fixed standard dose according to clinical guidelines. The inclusion of different tumor types in the two cohorts was intentional, as the primary objective of the study was not to compare tumor-specific outcomes, but to contextualize the clinical expression of SN-38-related toxicity under two distinct pharmacogenetic management strategies: genotype-guided dosing for irinotecan and fixed dosing without pharmacogenetic adjustment for SG.

Adult patients (≥18 years) with metastatic breast cancer treated with SG or with advanced gastrointestinal malignancies (predominantly colorectal and pancreatic cancer) treated with irinotecan were included in the study. Eligibility criteria required that patients had initiated treatment, had an interpretable *UGT1A1* genotype result, and had sufficient clinical follow-up to allow assessment of treatment-related toxicity. Patients who did not ultimately receive treatment, those with incomplete clinical records, or those who did not carry the UGT1A1*28 allele were excluded, as the analysis specifically aimed to explore the relationship between genotype and severe toxicity. This study was approved by the institutional Research Ethics Committee and conducted in accordance with the Declaration of Helsinki and applicable data protection regulations. All data were anonymised prior to analysis.

### 2.2. Clinical Variables Collected

Clinical, demographic, and oncological variables were retrospectively extracted from electronic medical records. Collected data included age, performance status (ECOG), estimated renal function, and relevant comorbidities, as well as tumour type, stage at diagnosis, sites of metastatic disease, and prior lines of systemic therapy.

With respect to treatment exposure, the standard dose of SG administered (10 mg/kg on days 1 and 8 of each 21-day cycle) [8] was recorded, together with any dose reductions, treatment delays, or treatment discontinuations applied due to toxicity.

For patients treated with irinotecan, the specific treatment regimen, initial dose administered, and any dose modifications implemented based on *UGT1A1* genotype were recorded. At our centre, irinotecan was prescribed in accordance with standard clinical practice guidelines. Irinotecan monotherapy was administered at a dose of 180 mg/m^2^ every 14 days, although standard dosing may range from 180 mg/m^2^ administered twice weekly to 350 mg/m^2^ every 21 days [18]. Combination regimens such as FOLFIRI and FOLFIRINOX included irinotecan at a dose of 180 mg/m^2^ every 14 days. In alternative monotherapy schedules, irinotecan was administered at doses of 150–180 mg/m^2^ every 21 days. Finally, liposomal irinotecan in combination with 5-fluorouracil and leucovorin was administered at a dose of 70 mg/m^2^ every 14 days, in accordance with international recommendations [29]. All genotype-guided dose adjustments were documented accordingly.

Treatment-related toxicity was assessed according to the Common Terminology Criteria for Adverse Events (CTCAE) version 5.0 [30]. Only grade ≥3 adverse events were recorded, with particular focus on toxicities typically associated with SN-38 exposure, including neutropenia, febrile neutropenia, diarrhoea, nausea, vomiting, severe asthenia, mucositis, and clinically significant elevations in liver enzyme levels. Toxicity-related hospitalisations and the cumulative duration of hospital stays were also documented.

### 2.3. UGT1A1 Genotyping Procedure

Genotyping of the UGT1A1*28 allele was performed using a KASP^®^ (Kompetitive Allele-Specific PCR) assay targeting the (TA)n_nn repeat polymorphism in the promoter region of the *UGT1A1* gene, corresponding to marker rs8175347, which discriminates between the UGT1A1*1 [(TA)6] and UGT1A1*28 [(TA)7] alleles. Biological samples consisted of saliva collected using sterile swabs. Genomic DNA was extracted using a standardised non-invasive isolation protocol and subsequently amplified by allele-specific PCR. Fluorescence signals generated by allele-specific probes enabled accurate assignment of the *1/*1, *1/*28, and *28/*28 genotypes, which were classified as normal, intermediate, or poor metabolisers according to the expected functional activity of *UGT1A1*.

In the SG cohort, as genotyping had not been performed prospectively, saliva samples were collected at the time patients developed severe toxicity. In contrast, for irinotecan-treated patients, *UGT1A1* genotyping was routinely performed as part of the standard pre-treatment assessment.

### 2.4. Patient Management and Clinical Evaluation

SG was administered in accordance with the approved summary of product characteristics at a dose of 10 mg/kg on days 1 and 8 of each 21-day cycle [8]. Dose modifications were applied based on individual clinical tolerance, as there are currently no official recommendations for *UGT1A1* genotype-guided dose adjustment. Toxicity management followed standard clinical practice.

In the irinotecan cohort, patient management incorporated genotype-guided dose adjustments in line with international pharmacogenetic recommendations from the DPWG [19]. Patients homozygous for UGT1A1*28 received initial dose reductions, while heterozygous UGT1A1*28 patients were managed on an individual basis according to observed toxicity and neutrophil counts. This cohort therefore served as a positive pharmacogenetic control, as dosing was personalised based on the expected metabolic capacity for SN-38. Toxicity was assessed according to the CTCAE version 5.0 criteria [30].

### 2.5. Economic Analysis

An economic analysis was conducted to estimate the direct healthcare costs associated with episodes of severe toxicity related to SN-38 exposure. For each patient, the duration of hospitalisation and the type of care unit required were recorded. Costs were calculated using the official tariffs of the Andalusian Health Service applicable during the study period, amounting to €771.43 per day for conventional medical ward hospitalisation and €1984.90 per day for intensive care unit admission [31]. The cost of *UGT1A1* genotyping using the KASP^®^ assay was estimated at €10.51 per test.

The primary objectives of the economic analysis were to estimate the average cost per treated patient and per hospitalised patient and to assess the economic feasibility of implementing pre-treatment genotyping prior to initiation of SG therapy.

### 2.6. Data Management and Statistical Analysis

Baseline characteristics, safety outcomes, and healthcare resource utilisation were summarised using descriptive statistics. Categorical variables are reported as counts and percentages, and continuous variables as medians with interquartile ranges, as appropriate. Given the observational nature of the study, the marked imbalance in cohort sizes, and the retrospective selection of the SG cohort based on the occurrence of severe toxicity, no formal statistical comparisons between treatment groups were performed. Multivariable analyses were not conducted due to the limited sample size of the SG cohort. Data management and descriptive analyses were performed using R software (version 4.3.2).

## 3. Results

### 3.1. Patient Flow and Baseline Characteristics

For this study, two independent cohorts were initially identified, comprising patients treated with SG or irinotecan in real-world clinical practice. In the irinotecan cohort, all adult cancer patients (≥18 years) treated between January 2021 and July 2025 who were carriers of the UGT1A1*28 allele, either in its heterozygous or homozygous form, were identified through the standard pre-treatment genotyping programme. Of the 113 patients initially screened, 38 did not ultimately initiate irinotecan therapy, and one additional patient began treatment in July 2025 but had insufficient follow-up and toxicity data available. After these exclusions, the final irinotecan cohort consisted of 74 patients with advanced gastrointestinal malignancies, predominantly colorectal and pancreatic cancer, all of whom were carriers of the UGT1A1*28 allele and had received at least one cycle of irinotecan.

In parallel, for the SG cohort, all patients with metastatic breast cancer treated between January 2024 and July 2025 who underwent retrospective *UGT1A1* genotyping after developing severe treatment-related toxicity (grade ≥3 according to CTCAE version 5.0 were identified [30]). All patients in this cohort were female, reflecting the real-world population treated with SG at our institution during the study period. A total of ten patients were genotyped; one patient was excluded after confirmation of a UGT1A1*1/*1 genotype (wild type for the *28 allele). The final SG cohort therefore comprised nine patients, all women with metastatic breast cancer treated with SG.

Both cohorts exhibited baseline characteristics typical of patients with advanced malignancies. Median age was comparable between groups, and the distributions of ECOG performance status, creatinine clearance, and tumour stage reflected metastatic or locally advanced disease in nearly all cases. The primary difference between cohorts arose from the genotyping strategy: in the irinotecan cohort, *UGT1A1* analysis was performed prior to treatment initiation to inform dosing decisions, whereas in the SG cohort, genotyping was conducted only after the development of severe toxicity, accounting for the high proportion of UGT1A1*28 carriers observed in this group. Complete baseline characteristics are presented in Table 1.

### 3.2. Incidence of Grade ≥3 Toxicity

Safety analyses focused on the occurrence of grade ≥3 adverse events, as defined by CTCAE version 5.0 criteria [30]. For descriptive purposes, adverse events were grouped into clinically relevant categories, including haematological toxicity (neutropenia, febrile neutropenia, thrombocytopenia, and anemia), gastrointestinal toxicity (nausea, vomiting, and diarrhea), asthenia/fatigue, neurological toxicity, hepatic toxicity, sepsis, and other serious toxicities not classified within the aforementioned categories.

A wide spectrum of serious adverse events was observed in the SG-treated cohort. Among haematological toxicities, several episodes of grade 4 febrile neutropenia were noted, some of which were complicated by infectious enterocolitis and septic shock, in addition to cases of grade 3 afebrile neutropenia and grade 3 thrombocytopenia. Episodes of moderate anemia, acute kidney injury, and hyponatraemia were also documented in the context of sepsis. Within the gastrointestinal toxicity profile, grade 3 nausea and vomiting were reported, as well as multiple cases of grade 3 diarrhoea. Systemic toxicity included grade 3 asthenia in several patients and grade 4 alopecia in one patient. One patient experienced acute-onset neurological toxicity, characterised by grade 3 severe headache, dizziness, light-headedness, and mental confusion, which markedly impaired daily functioning. Overall, all patients treated with SG experienced at least one grade ≥3 adverse event.

It is important to emphasise that the SG cohort included only patients who were retrospectively genotyped after developing severe toxicity. Consequently, the toxicity profile observed in this group does not reflect the overall incidence of adverse events among all patients treated with SG, but rather the phenotypic expression of toxicity in a population selected for clinical intolerance. This selection bias should be carefully considered when interpreting the frequency and distribution of the reported adverse events.

Grade ≥3 adverse events were also observed in the irinotecan cohort. The most frequent haematological toxicity was grade 3–4 neutropenia, with occasional cases of febrile neutropenia. Gastrointestinal adverse events included grade 3 diarrhoea and vomiting, and grade 3 asthenia was reported in a limited number of patients. Less frequent events, such as severe arterial hypertension or grade 3 elevations in liver enzyme levels, were also documented.

A descriptive overview of grade ≥3 treatment-related adverse events and hospital admissions in both treatment cohorts is provided in Table 2.

### 3.3. Hospital Admissions and Use of the Intensive Care Unit

Hospital admissions related to treatment-related toxicity were observed in both treatment cohorts. In the SG cohort, three patients (33.3%) required hospital admission due to severe adverse events. Admissions were primarily related to febrile neutropenia, severe diarrhoea, or septic complications.

In this cohort, a total of 25 hospitalisation days were recorded, including 5 days in the intensive care unit (ICU) and 20 days in conventional medical wards. Hospital stays were generally of moderate duration.

Hospital admissions were also documented in the irinotecan cohort, with eight patients (10.8%) requiring hospitalisation due to toxicity. A total of 68 hospitalisation days were recorded. Although fewer patients required admission, the cumulative duration of hospitalisation was longer, driven by several prolonged stays, including extended ICU admissions of 25 and 7 days. Overall, patients treated with irinotecan accounted for 32 ICU days and 36 days in medical wards.

ICU admissions were uncommon in both cohorts; however, prolonged ICU stays were observed in a small number of patients in the irinotecan cohort. A descriptive summary of hospitalisation burden and associated direct healthcare costs is provided in Table 3.

### 3.4. Economic Analysis of Toxicity

The analysis of direct healthcare costs associated with severe treatment-related toxicity was conducted using the official tariffs of the Andalusian Health Service (SAS), which are publicly available on its institutional website [31].

In the SG cohort, the total cost attributable to grade ≥3 toxicity events was €25,353.10, corresponding to a mean cost of €2817.01 per treated patient. Three of the nine patients (33.3%) required hospital admission, accounting for a total of 25 hospitalisation days, equivalent to an average of 2.78 hospital days per treated patient.

In the irinotecan cohort, the total cost associated with severe toxicity was €91,288.28, with a mean cost of €1233.63 per treated patient. Eight of the 74 patients (10.8%) required hospital admission, accumulating a total of 68 hospitalisation days and corresponding to 0.92 hospital days per treated patient. The cumulative duration of hospitalisation in this cohort was influenced by several prolonged intensive care unit stays, including admissions lasting 25 and 7 days.

When costs were analysed according to hospitalisation status, the mean cost per hospitalised patient was €8451.03 in the SG cohort and €11,411.04 in the irinotecan cohort. Conversely, when costs were averaged across all treated patients, higher mean costs were observed in the SG cohort, reflecting the higher frequency of severe toxicity requiring hospital care. A detailed breakdown of hospitalisation burden and direct healthcare costs is provided in Table 3.

### 3.5. Distribution of the UGT1A1*28 Allele and Its Association with Toxicity

In the SG-treated cohort, all patients included in the analysis (n = 9) were carriers of the UGT1A1*28 allele, either in the heterozygous (*1/*28) or homozygous (*28/*28) state. Genotyping was performed retrospectively following the onset of severe toxicity and confirmed that all carrier patients included in the study had experienced grade ≥3 adverse events according to CTCAE version 5.0 criteria [30]. The observed toxicities encompassed a broad clinical spectrum, including grade 4 febrile neutropenia, grade 3 diarrhoea, sepsis, severe asthenia, and, in some cases, neurological or hepatic toxicities. One patient who developed severe toxicity during the study period and was found to carry a UGT1A1*1/*1 genotype was identified but excluded from the analysis, as she did not carry the *28 allele.

In the irinotecan cohort, all patients were also carriers of the UGT1A1*28 allele, as this constituted an inclusion criterion for the study. Genotyping was systematically performed prior to treatment initiation, allowing genotype-guided dose adjustments in accordance with standard clinical practice. As a result, this cohort provides contextual information on the clinical expression of SN-38-related toxicity under pharmacogenetic-guided management.

Given the limited size of the SG cohort and the absence of an internal control group of non-carrier patients, no formal statistical analysis of the association between the *UGT1A1* genotype and toxicity was performed. Nevertheless, the observation that all UGT1A1*28 carriers included in the SG cohort developed grade ≥3 toxicity represents a clinically relevant descriptive signal within a population selected for treatment intolerance. These findings suggest a potential role of the *UGT1A1* genotype as a risk biomarker for SG-related toxicity, warranting further evaluation in prospective studies. Individual patient data and their relationship to toxicity are summarised in Table 2.

## 4. Discussion

The results of this study show that severe treatment-related toxicity (grade ≥3 according to CTCAE version 5.0 [30]) was frequently observed among patients treated with SG in real-world clinical practice. This observation is clinically relevant because, in the SG cohort, all patients who underwent *UGT1A1* genotyping after developing severe toxicity were carriers of the UGT1A1*28 allele. In contrast, in the irinotecan cohort, where pharmacogenetic-guided dose adjustment strategies were routinely applied, the proportion of patients experiencing severe toxicity was lower. Current pharmacogenetic guidelines, such as those from the DPWG [19], recommend initiating irinotecan at a reduced dose in patients homozygous for UGT1A1*28 and subsequently escalating based on tolerance. This contrast between a drug with well-established genotype-guided dosing recommendations (irinotecan) and another lacking such strategies despite sharing the same active metabolite (SG) highlights a relevant clinical context for exploring the role of pharmacogenetics in the management of SG-related toxicity.

Importantly, the toxicities observed in the SG cohort were not only frequent but also clinically severe. All patients experienced grade ≥3 adverse events, and one third required hospital admission, reflecting a substantial burden in terms of morbidity and healthcare resource utilization. These events included life-threatening complications such as febrile neutropenia and septic shock, underscoring that SG-related toxicity in this real-world context represents a clinically meaningful outcome rather than a transient or easily manageable adverse effect. In contrast, patients treated with irinotecan under a genotype-guided dosing strategy exhibited a lower frequency of severe toxicity and hospitalization. This comparison should be interpreted in a contextual rather than inferential manner, as it aims to highlight the clinical relevance of pharmacogenetic management for SN-38-based therapies rather than to directly compare drug- or tumor-specific toxicity profiles.

Overall, while both irinotecan and SG share SN-38-related toxicity, irinotecan benefits from established genotype-guided dosing strategies, whereas SG is currently administered without pharmacogenetic adjustment, which could increase the risk of severe toxicity and associated healthcare costs in susceptible patients.

When compared with previous research, our findings are consistent with the well-established association between UGT1A1*28 and severe SN-38-related toxicity described for irinotecan-treated patients, for whom genotype-guided dosing strategies have been shown to reduce the incidence of severe haematological toxicity [14,19]. However, evidence regarding the role of the *UGT1A1* genotype in patients treated with SG remains limited. Exploratory pharmacogenetic analyses from pivotal trials such as ASCENT and TROPiCS-02 included only a small number of UGT1A1*28 homozygous patients, potentially limiting their ability to detect genotype-driven safety signals [6,7]. However, our results are strongly supported by a recently published meta-analysis by Dello Russo et al. [32], which confirmed a significantly increased risk of grade ≥3 neutropenia and diarrhea in patients with the UGT1A1*28/*28 genotype treated with SG. Our real-world data highlight a striking overrepresentation of the UGT1A1*28 allele among patients experiencing severe SG-related toxicity, thereby extending previous findings and suggesting that this pharmacogenetic vulnerability may be more clinically apparent in routine practice where no genotype-guided dose adjustments are currently implemented.

Published evidence on irinotecan consistently demonstrates the clinically relevant impact of UGT1A1*28 on the risk of severe neutropenia, particularly in homozygous patients, thereby supporting the systematic implementation of genotyping as a therapeutic safety tool [33]. In addition, recent reviews including the systematic review conducted by our group on the influence of *UGT1A1* genotype on irinotecan dosing [14] provide robust evidence supporting personalised strategies to reduce toxicity associated with drugs that generate SN-38.

In this context, the findings observed in the SG-treated cohort are particularly noteworthy. The primary limitation of this study is the relatively small sample size of the SG cohort (n = 9) and the retrospective nature of the genotyping analysis. However, these findings must be interpreted within the current regulatory and clinical context, as SG has only recently received price and reimbursement approval in Spain [8]. Consequently, while the number of treated patients in real-world practice is still limited, the high severity of the toxicities observed in these initial cases, including grade 4 febrile neutropenia and septic shock, constitutes an early safety signal that cannot be ignored while larger cohorts are compiled. Although pivotal trials such as ASCENT [6] and TROPiCS-02 [7] established the efficacy of SG, their exploratory pharmacogenetic analyses included a very limited number of patients homozygous for UGT1A1*28, which may have underpowered the detection of genotype-driven safety risks. In contrast, our real-world series reveals a striking 100% prevalence of the UGT1A1*28 allele among patients experiencing severe intolerance, suggesting that the clinical expression of this vulnerability may be more pronounced in routine practice where no genotype-based dose adjustments are currently implemented.

From a pharmacological perspective, this finding is biologically plausible. Preclinical and translational studies have shown that sacituzumab govitecan releases SN-38 not only intracellularly but also systemically due to its hydrolytically labile linker, resulting in sustained exposure to the active metabolite and supporting the biological plausibility of *UGT1A1*-mediated toxicity [21]. Both irinotecan and SG release SN-38, whose clearance depends predominantly on *UGT1A1*-mediated glucuronidation [34]. Reduced or loss-of-function variants such as UGT1A1*28 diminish glucuronidation capacity, leading to increased systemic exposure to SN-38 and, consequently, a higher risk of haematological and gastrointestinal toxicity. The key difference lies in the fact that, whereas irinotecan dosing is adjusted according to genotype, SG is administered at a fixed dose (10 mg/kg), irrespective of the patient’s metabolic profile [8]. This may explain the more pronounced phenotypic expression of UGT1A1*28 associated toxicity observed in the SG cohort.

From an economic perspective, treatment with SG was associated with a higher mean cost per treated patient, primarily driven by a greater proportion of patients requiring hospital admission. It is important to note that the economic comparison between cohorts reflects two distinct management strategies. The costs observed in the SG cohort are representative of a ‘failure-to-prevent’ scenario (treating established severe toxicity), whereas the Irinotecan cohort reflects a ‘prevention’ scenario. Given that the direct cost of *UGT1A1* genotyping is minimal (€10.51) compared to the substantial economic burden of a single toxicity-related hospitalization (€771.43–€1984.90/day [31]), our data underscore the potential economic implications of identifying patients at increased risk. Our group’s previous experience has demonstrated that the systematic implementation of pharmacogenetics in oncology yields both clinical and economic benefits [35] by reducing avoidable toxicity and optimising healthcare resources. Although a formal cost-effectiveness or cost-utility analysis comparing different genotyping strategies (universal, reactive, or no testing) was beyond the scope of this exploratory real-world study, these findings provide relevant preliminary evidence to inform such evaluations and highlight the potential value of preventive *UGT1A1* testing in SG-treated patients. Future prospective studies incorporating formal health economic modeling will be required to determine the most efficient testing strategy.

From a translational perspective, the findings of this study support consideration of *UGT1A1* genotyping as a potential tool to optimize the safe use of SG in routine clinical practice. Unlike irinotecan, for which pharmacogenetic testing is already integrated into clinical guidelines, SG is currently administered without genotype-guided dose adjustment. Potential barriers to implementation include limited clinician awareness of the pharmacogenetic implications of SN-38-based therapies, variability in access to rapid genotyping, and the absence of regulatory or reimbursement frameworks supporting routine testing. Nevertheless, the low cost and wide availability of *UGT1A1* genotyping, together with the substantial clinical and economic burden associated with severe SG-related toxicity, suggest that pre-emptive testing could be feasibly incorporated into clinical workflows. Importantly, these findings should inform future prospective studies and regulatory discussions rather than immediate changes in clinical practice.

However, this study has several limitations. The retrospective genotyping strategy applied in the SG cohort introduces an inherent selection bias, as only patients who developed severe toxicity underwent pharmacogenetic testing. This design may lead to an overestimation of the association between UGT1A1*28 and treatment intolerance, as well as of the incidence of severe toxicity and associated healthcare costs. This approach reflects current real-world practice, as no genotype-guided dosing recommendations are available for SG and treatment is initiated at a fixed standard dose. In this context, the early onset of severe toxicity after treatment initiation and the subsequent clinical improvement following dose interruption or discontinuation support a temporal relationship between SG exposure and the observed adverse events. Although potential confounding factors such as comorbidities, concomitant medications, and prior lines of therapy were not formally controlled for due to the limited sample size, these findings should be interpreted as hypothesis-generating and intended to inform future prospective studies rather than universal clinical recommendations. A further limitation is the marked imbalance in sample size between the two cohorts, as well as the inclusion of different tumour types; however, the primary objective was not to compare tumour-specific outcomes but to contextualize the clinical expression of SN-38-related toxicity under different pharmacogenetic management strategies. The observed sex imbalance between cohorts should also be interpreted in light of disease epidemiology and sample size. Finally, the lack of pharmacokinetic data precluded direct correlations between drug exposure and toxicity. Despite these constraints, this study highlights a clinically relevant pharmacogenetic signal in a setting where no genotype-guided recommendations currently exist.

## 5. Conclusions

In this study, severe toxicity associated with SG in real-world clinical practice was observed in patients carrying the UGT1A1*28 allele who were genotyped after the development of severe adverse events. Our results highlight a critical clinical and economic disparity: the cost of *UGT1A1* genotyping is substantially lower (€10.51) than the cost of a single hospitalization episode (up to €1984.90/day). In a setting where SG and irinotecan share the same active metabolite (SN-38), the absence of pharmacogenetic-guided dose recommendations for SG stands in stark contrast to the established management of irinotecan. Taken together, our findings suggest that the UGT1A1*28 allele plays a clinically relevant role in the safety profile of SG. Although limited by sample size, this consistent pharmacogenetic signal supports the urgent need for prospective studies to evaluate genotype-guided interventions, such as preventive dose adjustment or intensified monitoring, to improve treatment tolerability and healthcare resource utilization in patients receiving Sacituzumab Govitecan.

## Figures and Tables

**Table 1:** Baseline characteristics of the study population according to treatment cohort.

	Sacituzumab-Govitecan (N = 9)	Irinotecan (N = 74)
**Sex (%)**		
F	9 (100)	26 (35.1)
M	0 (0)	48 (64.9)
**Age, median (IQR)**	61 (54–62)	63.5 (60–71)
**Creatinine clearance, median (IQR), mL/min**	93.3 (89.9–102.1)	85.16 (75.53–104.10)
**Ethnicity (%)**		
**European**	9 (100)	74 (100)
**ECOG** **^a^** **(%)**		
0	3 (33.3)	20 (27.0)
1	6 (66.7)	43 (58.1)
2	0 (0)	11 (14.9)
**Stage of cancer (%)**		
I–III	0 (0)	12 (16.2)
IV	9 (100)	62 (83.8)
**Tumor location (%)**		
Breast	9 (100)	0 (0)
Colorectal	0 (0)	33 (44.6)
Pancreas	0 (0)	36 (48.6)
Others	0 (0)	5 (6.8)
**Conventional chemotherapy regimens (%)**		
Sacituzumab-govitecan monotherapy	9 (100)	0 (0)
Irinotecan monotherapy	0 (0)	8 (10.8)
FOLFIRI ^b^	0 (0)	34 (45.9)
FOLFIRINOX ^c^	0 (0)	33 (44.6)
UGT1A1*28 **^d^** **Genotype (%)**		
UGT1A1*1/*28	4 (44.4)	55 (74.3)
UGT1A1*28/*28	5 (55.6)	19 (25.7)

^a^. ECOG: Eastern Cooperative Oncology Group performance status. ^b^. **FOLFIRI**: chemotherapy regimen consisting of **FOL**inic acid (leucovorin), **F**luorouracil (5-FU), and **IRI**notecan. ^c^. **FOLFIRINOX**: regimen composed of **FOL**inic acid (leucovorin), **F**luorouracil (5-FU), **IRI**notecan, and **OX**aliplatin. ^d^. *UGT1A1* genotyping focused on the TA-repeat polymorphism **rs8175347**, in the promoter region, which defines the UGT1A1 *28 allele (TA7).

**Table 2:** Summary of Grade ≥3 treatment-related adverse events according to UGT1A1*28 genotype.

Adverse Event Category	Sacituzumab-Govitecan (N = 9)	Irinotecan (N = 74)
Any grade ≥3 adverse event. No. of Patients-No (%)	9 (100)	28 (37.8)
Hematologic toxicity	7 (77.8)	15 (20.3)
Digestive toxicity	5 (55.6)	8 (10.8)
Asthenia/fatigue	4 (44.4)	6 (8.1)
Others	5 (55.6)	2 (2.7)
**Hospital admission due to toxicity No (%)**		
Intensive Care Unit (ICU)	1 (11.1)	2 (2.7)
Medical Oncology Ward	2 (22.2)	6 (8.1)

Only grade ≥3 treatment-related adverse events are shown, according to CTCAE v5.0 criteria. Percentages are calculated over the total study population. Data are presented descriptively; no formal statistical comparisons were performed.

**Table 3:** Hospitalisation burden and direct healthcare costs associated with treatment-related toxicity by treatment group.

Variable	Sacituzumab-Govitecan (N = 9)	Irinotecan (N = 74)
Patients requiring hospital admission due to treatment-related toxicity, n (%)	3 (33.3)	8 (10.8)
Total hospitalization days	25	68
Hospital days per treated patient, mean	2.78	0.92
ICU days (cost 1984.90 €/day)	5	32
Medical ward days (cost 771.43 €/day)	20	36
Total hospitalization cost (€)	€25,353.10	€91,288.28

Data are presented descriptively. No formal statistical comparisons between treatment cohorts were performed due to differences in cohort size and clinical context.

## Data Availability

The raw data supporting the conclusions of this article will be made available by the authors on request.

## Abbreviations

The following abbreviations are used in this manuscript:

ADC	Antibody–Drug Conjugate
APC	Article Processing Charge
CES1	Hepatic carboxylesterases
CES2	Hepatic carboxylesterases
C_max	Maximum plasma concentrations
CPIC	Clinical Pharmacogenetics Implementation Consortium
CTCAE	Common Terminology Criteria for Adverse Events
DPWG	Dutch Pharmacogenetics Working Group
ECOG	Eastern Cooperative Oncology Group
EMA	European Medicines Agency
FDA	U.S. Food and Drug Administration
HER2	Hormone receptor and human epidermal growth factor receptor 2
ICU	Intensive Care Unit
IQR	Interquartile Range
mTNBC	Triple-negative metastatic breast cancer
KASP^®^	Kompetitive Allele-Specific PCR
PK	Pharmacokinetics
SAS	Andalusian Health Service (Servicio Andaluz de Salud)
SEFF	Spanish Society of Pharmacogenomics and Pharmacogenetics (Sociedad Española de Farmacogenómica y Farmacogenética)
SG	Sacituzumab Govitecan
SN-38	7-ethyl-10-hydroxycamptothecin
SN-38G	SN-38 glucuronide
TROP-2	Trophoblast cell-surface antigen 2
UGT1A1	Uridine diphosphate glucuronosyltransferase 1 family, polypeptide A1
